# Access to Social Protection by People Living with, at Risk of, or Affected by HIV in Eswatini, Malawi, Tanzania, and Zambia: Results from Population-Based HIV Impact Assessments

**DOI:** 10.1007/s10461-022-03645-1

**Published:** 2022-03-22

**Authors:** David Chipanta, Audrey Pettifor, Jessie Edwards, Danielle Giovenco, Hillary Mariko Topazian, Rachel M. Bray, Monique C. Millington, Janne Estill, Olivia Keiser, Jessica E. Justman

**Affiliations:** 1 Joint United Nations Programme on HIV/AIDS (UNAIDS), 20 Avenue appia, 1211 Geneva, Switzerland; 2 Institute of Global Health, University of Geneva, Geneva, Switzerland; 3 Department of Epidemiology, Carolina Population Center, University of North Carolina-Chapel Hill, Chapel Hill, USA; 4 ICAP at Columbia, Mailman School of Public Health, Columbia University, New York, USA; 5 Departments of Epidemiology and Medicine, Columbia University, New York, USA

**Keywords:** Social protection, Cash transfers, People living with HIV, Sex workers, Adolescent girls and young women, Men who have sex with men

## Abstract

We aimed to measure social protection coverage among the general population, women and men living with HIV (WLHIV, MLHV), female and male sex workers (FSW, MSW), men who have sex with men (MSM), adolescent girls young women (AGYW), and orphans vulnerable children (OVC) in Eswatini, Malawi, Tanzania, and Zambia. We used Population-Based HIV Impact Assessment data. We operationalised social protection benefits as external economic support from private and public sources to the household in the last three or 12 months. We estimated survey-weighted proportions and 95% confidence intervals (CI) for each population receiving social protection benefits. The sample size ranged from 10,233 adults ages 15–59 years in Eswatini to 29,638 in Tanzania. In the surveyed countries, social protection coverage among the general population was lower than the global average of 45%, ranging from 7.7% (95% CI 6.7%–8.8%) in Zambia to 39.6% (95% CI 36.8%–42.5%) in Eswatini. In Malawi and Zambia, social protection coverage among OVC, AGYW, SW, MSM, and people living with HIV (PLHIV) was similar to the general population. In Eswatini, more AGWY reported receiving social projection benefits than older women and more men not living with HIV reported receiving social protection benefits than MLHIV. In Tanzania, more WLHIV than women not living with HIV, MLHIV than men not living with HIV, and FSW than women who were not sex workers reported receiving social protection benefits. More data on access to social protection benefits by PLHIV or affected by HIV are needed to estimate better their social protection coverage.

**Supplementary Information:**

The online version contains supplementary material available at 10.1007/s10461-022-03645-1.

## Introduction

In 2016, the United Nations Member States adopted the Political Declaration on Ending AIDS by 2030. The declaration laid out commitments to reduce new HIV infections to fewer than 500,000, AIDS-related deaths to less than 500,000 and eliminate HIV-related stigma and discrimination globally [1]. Summarized in the Ten UNAIDS Fast-Track Commitments, if achieved by 2020, the world would be on course to ending AIDS as a public health threat by 2030 [1, 2]. With the COVID-19 pandemic risking setting back progress against HIV, effectively implementing and measuring these commitments is crucial to the AIDS response [3, 4]. Most of the Ten UNAIDS Fast-Track Commitments have been measured. However, Commitment 6, “Ensure that by 2020, 75% of people living with, at risk of or affected by HIV benefit from HIV-sensitive social protection,” has not. Social protection is being prioritized as a vital policy tool for increased health and development by policymakers. The UNAIDS Strategy 2021–2026 focuses on ending inequalities in HIV prevention and treatment outcomes. It has included social protection as an area of work, arguing that social protection advances the AIDS response by addressing inequalities, which drive the HIV epidemic [5, 6]. Social protection is also integral to the COVID-19 pandemic response. More than 3,330 new social protection programmes worth US$2.9 trillion have been introduced globally since 2020 to mitigate the health, social and economic fallout from the COVID-19 pandemic [7]. More than cash transfers, social protection comprises public transfers, and policies, such as social safety nets, social security and labour market policies to help people manage risk and protect them from poverty and destitution [8, 9]. People living with HIV (PLHIV), key populations [sex workers (SW), gay men and other men who have sex with men (MSM), people who inject drugs, transgender people and prisoners), adolescent girls and young women (AGYW), orphans and vulnerable children (OVC) and others have increasingly demanded access to social protection benefits [10, 11].

Social protection impacts can be powerful, especially in settings where people face multiple threats to their health and well-being. It tackles the structural determinants of health, including monetary poverty, lack of education, unemployment, low savings, disempowerment, low child immunization and birth registrations [12–14]. It also improves the psychological wellbeing and increases the quantity and quality of food consumed by beneficiaries [15, 16]. Social protection impacts HIV prevention and treatment outcomes through complex pathways [17–20]. It enables people to withstand life shocks, empowering them to reduce dependence on HIV risky coping strategies [21–24]. HIV infection in women is strongly associated with physical and emotional violence and male controlling behaviour [25]. Cash transfers reduce intimate partner violence, which is associated with HIV infection, among beneficiaries by removing poverty-related stress, reducing conflicts arising from tight household budgets and empowering women [26]. Social protection helps people overcome financial and other barriers to HIV treatment and prevention services, reducing inequity in accessing and using these services [22, 24, 27]. By helping enrol and keep adolescent girls and young women in school, social protection protects them from HIV [17, 28, 29]. It is also critical for unpaid caregivers, many of whom are women [24]. Social protection policies and laws, including workplace policies, uphold the rights to gainful employment, social security, housing, non-discrimination, and other universal social and economic rights [30, 31].

Several features of social protection implementation in sub-Saharan Africa are crucial to HIV prevention and treatment efforts. First, countries, including those heavily affected by HIV, are scaling up social protection programmes to help people overcome shocks such as climate change, price increases, and most recently COVID-19 pandemic [7, 32]. Shocks interrupt HIV services [4]. Pandemic related social protection programmes help mitigate the vulnerability of people living with, at risk of or affected by HIV. Second, cash transfers are a primary delivery mechanism of social protection [9, 33]. Because of their multiple impacts on health and development domains, cash transfers have gained heightened attention in HIV prevention and treatment efforts [22, 34–36]. Third, children, adolescents, girls and women, people with disabilities, and older people, are often the focus populations of social protection programmes [9, 33]. These population groups are also affected by HIV. Fourth, social protection, including floors, is promoted as a human right, applicable to everyone, including people living with, at risk of or affected by HIV [30, 31]. Thus, measuring social protection coverage is crucial for the AIDS response.

In 2017 the UNAIDS National Composite and Policy Index (NCPI) first reported data on social protection [37]. These NCPI social protection data reflect whether social protection strategies are HIV sensitive, that is, whether they refer to HIV or recognize PLHIV, key populations, AGYW, OVC and people affected by HIV as crucial beneficiaries; and whether they address unpaid work in the HIV context [38]. However, none of these elements measure Commitment 6 directly. This study aimed to measure Commitment 6 by estimating social protection coverage among the general population and seven sub-population groups who are living with, at risk of or affected by HIV: women and men living with HIV (WLHIV, MLHIV), female and male sex workers (FSW, MSW), MSM, AGYW and OVC. We estimated social protection coverage among these groups using publicly available Population-Based HIV Impact Assessment (PHIA) data from four high HIV prevalence countries: Eswatini, Malawi, Tanzania and Zambia.

## Methods

### Study Setting

Table 1 displays selected economic indicators, HIV estimates, and the proportion of people who reported receiving social protection benefits in the four study countries. The countries are located in Eastern and Southern Africa, the epicentre of the HIV epidemic. Eswatini, Tanzania and Zambia are lower middle-income countries. Malawi is a low-income country. Eswatini and Zambia are among the countries with the highest HIV prevalence and incidence worldwide. Of the four countries, they are also the most unequal in terms of wealth. Their Gini coefficients, a measure of inequality, were 54.6 and 57.1, respectively, on a scale of zero (total equality) to 100% (full inequality) [39]. All four countries have established national social protection programmes and mature HIV epidemics [8, 40].

**Table 1:** Per capita gross domestic product, Gini index, poverty ratio, HIV estimates and proportion of people who reported accessing any social protection benefit, by country

Indicator	Eswatini	Malawi	Tanzania	Zambia
Population^a^	1,148,130	18,628,747	58,005,463	17,86, 030
Per capita gross domestic product (GDP) (current USD)^a^	3894.68	411.55	1122.12	1305.06
Gini index (%. 0 represents perfect equality, 100 perfect inequality)^a^	54.6	44.7	40.5	57.1
Number of adults and children living with HIV Estimated number with 95% confidence intervals)^b^ (1000 s)	200 [190 – 220]	1 100 [960–1 100]	1 700 [1 500–1 800]	1 200 [1 200– 1 300]
HIV prevalence of adults ages 15 to 49 (Estimated percentage of population living with HIV with 95% confidence intervals)^b^	27.0 [24.6–28.7]	8.9 [7.6–9.6]	4.8 [4.1–5.3]	11.5 [10.9–12.1]
HIV incidence per 1000 population (adults 15–49) (Estimated incidence with 95% confidence intervals)	9.77 [7.79–12.44]	3.71 [3.13–4.23]	2.57 [2.27–2.87]	6.03 [5.12–7.28]
HIV testing and treatment cascade				
Percentage of PLHIV who know their status (Estimated percentage with 95% confidence intervals)^b^	> 95% [91– > 95]	90% [81–95]	83% [75–90]	90% [85– > 95]
Percentage of PLHIV who are on antiretroviral therapy (Estimated percentage with 95% confidence intervals)^b^	> 95% [88– > 95]	79% [71–84]	75% [67–81]	85% [80–92]
Percentage of PLHIV who have suppressed viral loads (Estimated percentage with 95% confidence intervals)^b^	92% [85– > 95]	72% [65–77]	69% [62–74]	77% [72–82]
SW population size estimate (#)^b^	4000	36,400	155,500	18,000
MSM population size estimate (#)^b^	2400	42,600	49,700	6500
Social protection coverage in at least one area (percentage)^c^	–	21.3	–	15.3

^a^World Bank Population and GDP per capita (2019); GINI index for Eswatini (2016), Malawi (2015), Tanzania (2017) and Zambia (2015); poverty head count data for Eswatini (2015), Malawi (2016), Tanzania (2018) and Zambia (2015) (https://data.worldbank.org/indicator/SP.POP.TOTL?locations=SZ-MW-TZ-ZM)

^b^AIDSinfo [Internet]. Geneva: Joint United Nations Programme on HIV/AIDS (https://aidsinfo.unaids.org/)

^c^International Labour Organization (ILO). World social protection report 2017–2019: Universal social protection to achieve the Sustainable Development Goals. Geneva: ILO; 2017. Areas include child and family benefits, unemployment support, and health protection

– Data not available

As of 2020, Eswatini had fewer PLHIV (200,000) than Malawi (1,100,000), Tanzania (1,700,000) or Zambia (1,200,000) [40]. Tanzania had the largest estimated number of SW (155,500) and MSM (49,700), followed by Malawi (36,400 SW; 42,600 MSM), Zambia (18,000 SW; 6500 MSM) and Eswatini (4000 SW; 2400 MSM) [40].

### Data Sources

We used Indicator 1.3.1, Proportion of population covered by at least one social protection benefit, of the Sustainable Development Goals (SDGs) [41], which has been identified by UNAIDS as a proxy to measure Commitment 6.

We analysed data from the PHIA surveys that were publicly available and had data on social protection coverage among OVC, AGYW, PLHIV, SW and MSM. The PHIA surveys measured the impact of HIV programs in countries supported by the United States President’s Emergency Plan for AIDS Relief. These cross-sectional surveys were administered to consenting individuals in nationally representative random cluster samples of households. Study procedures included administering questionnaires, household-based HIV counselling and testing, and immediate return of point of care test results. The surveys assessed HIV status and included questions about external economic support and engagement in sex work for men and women. They identified AGYW ages 15 to 24 years directly, and indirectly, MSM and OVC status through behavioural and family demographic questions. Data on HIV and social protection variables were completed and available for four surveys, allowing for standardized measures across the corresponding countries [42]: Eswatini (2016–2017), Malawi (2015–2016), Tanzania (2016–2017) and Zambia (2016). We obtained the PHIA data sets from the PHIA Project website at https://phia-data.icap.columbia.edu/datasets.

We used the Household, Adult and Child Interview and Adult HIV Biomarker data sets.

In participating households, a household questionnaire was administered to the household head, who indicated all individuals living in the household (referred to as the roster or household list). Then, individual questionnaires were administered to eligible and consenting individuals in the household. Adults (15 years and older) completed an adult questionnaire. Adults also provided data on their children ages 0–14 years as part of the “children” module of the adult questionnaire. The Adult HIV Biomarker data set contained laboratory confirmed HIV test results of all adults and adolescents aged 15 and older who completed an individual interview and consented or assented to provide blood samples for HIV testing. The child interview data set included variables from the roster, such as age and gender, and questions from the adult questionnaire’s children module that were attached to the child’s records [42].

### Variables and Outcome Descriptions

We included women and men (15 to 59 years old) who were interviewed. We defined the respondent as HIV positive if their HIV biomarker test was positive. SW were defined as males or females aged 15 years or more who reported selling sex for money in the past 12 months; and AGYW were defined as females 15–24 years of age. We defined a person as MSM if the respondent was male and their first, second or third most recent sexual partner in the last 12 months was male. We also included OVC, defined as children ages 0–17 years who were orphaned, or HIV-positive, lived in a household with chronically ill parents or experienced a recent death from chronic illness. If gender was missing, the person was excluded from the analysis. Analyses of adults included only those adults interviewed, whereas all children in the roster ages 0–17 years were eligible for inclusion if the household head indicated that they were OVC.

We defined social protection as any external economic support from private and public sources to the household in the last three or 12 months. Our inclusion of private transfers in our operationalisation of social protection deviates from the World Bank and ILO's definitions of social protection, which focus on public transfers. We recorded the receipt of any child support provisions if the respondent acknowledged receiving any support, including school, social, material, emotional or medical support. (See Supplementary Table 1 in Appendix 1 for details on how the variables were coded).

### Analysis

We estimated the proportion receiving any social protection benefits by using the SAS survey means procedure to determine the weighted proportion of persons who reported receiving any social protection benefit for the identified population groups. Survey weights accounting for non-response using Chi-squared automatic interaction detector (CHAID) analysis, non-coverage and the probability of selection were applied. We used individual interview weights in the analyses of adults and household weights in OVC analyses. Variances and 95% confidence intervals (CI) were estimated using the corresponding jackknife replicate weights [43].

One-sample t-tests were used to test whether survey estimates differed significantly from the global average of 45% social protection coverage [8]. Rao-Scott Chi-square tests of association accounting for the complex sample design were used to indicate whether social protection access differed between WLHIV and not WLHIV, MLHV and not MLHIV, FSW and not FSW, MSW and not MSW, MSM and not MSM, AGYW and women 25 years or older. We set the statistical significance level α = 0.10. We used SAS v9.4 for the analyses [44]. The code is given in Appendix 2.

## Results

Table 2 shows the sample distribution by country and population groups. The sample percentages are unweighted. We did not aggregate results within and across countries due to the different sampling weights used. The sample comprised 10,233 adults ages 15–59 years in Eswatini, 19,106 in Malawi, 29,638 in Tanzania and 21,278 in Zambia, along with 2573 OVC ages 0–17 years in Eswatini, 4471 in Malawi, 7,388 in Tanzania and 6094 in Zambia. The AGYW population groups comprised between 19.7% and 21.6% of the adult sample in each country. OVC accounted for between 19.1% (Malawi) and 27.8% (Eswatini) of children ages 0–17. MSM and SW accounted for less than 1% of the sample in each country, except in Tanzania, where FSW made up 2.7%. We did not report estimates for MSM and SW for Eswatini because there were fewer than 25 observations.

**Table 2:** Sample distribution by country and population group, unweighted percentage (%) and size, PHIA

Population group	Eswatini (2016–2017)		Malawi (2015–2016)		Tanzania (2016–2017)		Zambia (2016)	
	%	n	%	n	%	n	%	n
Adults ages 15–59 years								
PLHIV—female^a^	20.1	1918	8.8	1477	4.2	1192	8.8	1688
PLHIV—male^a^	9.1	870	4.1	680	1.8	517	4.1	779
MSM	–	–	0.8	161	0.2	67	0.2	51
SW—female	–	–	1.0	200	2.7	803	0.4	76
SW—male	–	–	0.2^b^	32	0.4	133	0.1^b^	31
AGYW	19.7	2013	21.5	4102	20.3	6031	21.6	4587
Totals	100.0	10,233	100.0	19,106	100.0	29,638	100.0	21,278
Children ages 0–17 years								
OVC	27.8	2573	19.1	4471	20.3	7388	22.0	6094
Total	100.0	9271	100.0	23,432	100.0	36,376	100.0	27,655

^a^Denominators for PLHIV percentages were 9,556 in Eswatini, 16,698 in Malawi, 28,347 in Tanzania and 19,113 in Zambia, and excluded people who did not test for HIV or received indeterminate HIV test results

^b^Estimate based on 25–49 persons/observations and should be interpreted with caution

Estimates of unweighted survey proportions with sample size

– Results had fewer than 25 adults identified during the survey and were suppressed

Among the general population, the proportion who reported receiving any social protection benefits ranged from 7.7% (95% CI 6.7%–8.8%) in Zambia to 39.6% (95% CI 36.8%–42.5%) in Eswatini (Table 3). The proportion reporting receiving social protection benefits was significantly lower than the 2017–2019 global average of 45% in all population groups, except among OVC (t = 0.11, p = 0.91) and AGYW (t = 0.12, p = 0.90) in Eswatini, where it was not different from the global social protection average. The proportion reporting access to social protection benefits did not differ between AGYW and women 25 years or older, SW and not SW, MSM and not MSM, PLHIV and not PLHIV in Malawi and Zambia. In Eswatini and Tanzania the proportion reporting receiving social protection benefits was similar across sub-population groups, with a few exceptions. In Eswatini, 44.8% of AGYW reported receiving social protection benefits compared to 37.5% of older women aged 25–59 (χ^2^ = 28.6, p < 0.001). Fewer MLHIV reported receiving social protection benefits than men not living with HIV (37.3% versus 40.7%, χ^2^ = 3.0, p = 0.08). In Tanzania, 13.6% of WLHIV compared to 9.2% of women not living with HIV reported receiving social protection benefits (χ^2^ = 15.7, p < 0.001). More MLHIV than men not living with HIV reported receiving social protection benefits (10.8% versus 8.0%, χ^2^ = 15.7, p < 0.001). More female sex workers reported receiving social protection benefits than women who were not SW (11.7% versus 9.3%, χ^2^ = 3.2, p = 0.08) but fewer AGYW reported receiving social protection benefits than older women aged 25–59 (8.7% versus 9.9% χ^2^ = 3.9, p = 0.05) (Table 3).

**Table 3:** Proportion reporting any external economic support, last 12 months by country and population group, PHIA (%, 95% CI, sample size)

Population	Eswatini (2016–2017)^a,c^				Malawi (2015–2016)^a^				Tanzania (2016–2017)^a^				Zambia (2016)^a^			
Total population ages 15–59	39.6	[36.8, 42.5]	10,233	–	14.6	[13.4, 15.9]	19.106	–	8.8	[7.9, 9.7]	29.638	–	7.7	[6.7, 8.8]	21.278	–
PLHIV—female	39.4	[35.7, 43.1]	1918	1.6 [0.2]	14.5	[12.2, 16.8]	1477	0.3 [0.6]	13.6	[10.8, 16.5]	1192	15.7 [< 0.001]	7.3	[5.7, 8.8]	1.688	0.9 [0.34]
Not PLHIV—female	42.0	[38.2, 45.0]	3616		15.0	[13.7, 16.6]	8267		9.2	[8.2, 10.1]	14,886		8.0	[6.8, 9.3]	9.284	
PLHIV- male	37.3	[32.8, 41.8]	870	3.0 [0.08]	14.8	[11.5, 18.0]	680	0.0 [0.99]	10.8	[7.1, 14.4]	517	3.0 [0.08]	6.4	[4.5, 8.3]	779	1.9 [0.17]
Not PLHIV—male	41.0	[37.5, 43.9]	3152		15.0	[13.2, 16.3]	6274		8.0	[7.1, 8.9]	11,752		7.8	[6.7, 9]	7.362	
MSM	–	–	–	–	11.8	[6.5, 17.1]	161	0.9 [0.33]	9.8	[0.0, 20.0]	67	0.1 [0.71]	7.0	[0.4, 13.7]	51	0.0 [0.87]
Not MSM—male	–	–	–	–	15.0	[13.1, 16]	7846		8.1	[7.2, 9.1]	12,822		7.6	[6.5, 8.7]	9.119	
SW—female	–	–	–	–	12.9	[7.4, 18.4]	200	0.5 [0.5]	11.7	[8.5, 14.9]	803	3.2 [0.08]	6.4	[0.6, 12.3]	76	0.2 [0.65]
Not SW—female	–	–	–	–	15.0	[13.6, 16.1]	10,899		9.3	[8.3, 10.3]	15,946		7.8	[6.7, 9.0]	12.032	
SW—male	–	–	–	–	4.2^d^	[0.0, 13.1]	32	2.0 [0.15]	7.8	[1.8, 13.8]	133	0.0 [0.91]	11.9^d^	[0.0, 25.9]	31	0.7 [0.42]
Not sw—male	–	–	–	–	15	[13.1, 16]	7975		8.1	[7.2, 9.1]	12,756		7.6	[6.5, 8.7]	9.139	
AGYW	45.0	[41.0, 48.6]	2013	28.6 [< 0.001]	15.0	[13.2, 16.7]	4102	0.1 [0.72]	8.7	[7.5, 10.0]	6031	3.9 [0.05]	7.9	[6.5, 9.3]	4.587	0.1 [0.78]
Women age 25+	38.0	[34.5, 40.5]	3831		15.0	[13.4, 15.9]	6997		9.9	[8.8, 11]	10,718		7.8	[6.6, 8.9]	7.521	
OVC^b^	44.8	[41.5, 48.1]	2573	–	17.4	[15.5, 19.3]	4471	–	6.1	[4.9, 7.3]	7388	–	14.4	[12.3, 16.4]	6.094	–

^a^Combined external economic support to the household in the last three or 12 months: social pension, material or financial support for shelter, food assistance provided at the household or external institution, income generation support in cash or kind (e.g. agricultural inputs), material support for education (e.g. uniforms, school books, education, tuition support, bursaries), assistance for school fees, cash transfer (e.g. pension, disability grants, child grant) or other. Denominator: all interviewed adults ≥ 15 years included in key population group definitions. Numerator: those who indicated social protection coverage

^b^Combined school, social, material, emotional and medical support. Denominator: children < 18 years old, conditional on whether the child, natural mother, and/or natural father has been very sick for at least three months during the past 12 months (too sick to work or do normal activities). Numerator: those who indicated receipt of child support in the last 12 months

^c^No 12-month variable included in the data set

^d^Estimate based on 25–49 persons/observations and should be interpreted with caution. Results presented are estimates of proportions with 95% confidence intervals and sample size

– Results had fewer than 25 adults identified during the survey and were suppressed

## Discussion

This study found wide variation in the proportion who reported receiving social protection benefits by population group. The proportion who reported receiving social protection benefits was lower than the 2017–2019 global average of 45% [8] in all population groups, except for OVC and AGYW in Eswatini. Commitment 6 may have been too ambitious. AGYW, SW, MSM and PLHIV reported receiving social protection benefits similar to other individuals in Malawi and Zambia. In Tanzania, more WLHIV and FSW reported receiving social protection benefits than women not living with HIV and women who were not sex workers. Fewer AGYW reported receiving social protection benefits than women 25 years or older. In Eswatini, more AGYW reported receiving social protection benefits than women 25 years or older. However, fewer MLHIV reported receiving social protection benefits than men not living with HIV.

The finding that the proportion of the general population reporting receiving any social protection benefit was lower than the global average and varied widely, from 7.7% (95% CI 6.7–8.8) in Zambia to 39.6% (95% CI 36.8–42.5) in Eswatini, is consistent with existing evidence. The International Labour Organization (ILO) estimated that only 45% of the global population accessed at least one social protection benefit as of 2019, whereas only 17.8% of Africans were estimated to be covered by social protection. About one fifth (21.3%) of the total population in Malawi, 15.3% in Zambia, and 86% and 3.2% of older people (persons above statutory retirement age) in Eswatini and Tanzania, respectively, accessed social protection services based on ILO data [8]. In our study, only Eswatini exceeded the Africa regional average social protection coverage of 17.8%. Malawi met the Africa regional average social protection coverage among male PLHIV, and OVC. Tanzania and Zambia did not. Except for AGYW and OVC in Eswatini, the proportion that reported receiving social protection benefits for all countries examined was below the global social protection coverage of 45%.

Based on these findings, Commitment 6 may have been too ambitious. For example, none of the countries in our study reported any population group accessing social protection benefits more than the 2017–2019 global average of 45%, let alone 75% stipulated in Commitment 6. However, the 2016 Political Declaration from which the Ten UNAIDS Fast-Track commitments are derived stated: “… 75 per cent of people living with, at risk of and affected by HIV *who are in need* [Italics added for emphasis] benefit from HIV-sensitive social protection…” [1] It focused on a subset of people living with at risk of or affected by HIV in “need” of social protection benefits; not all people living with at risk of or affected by HIV reflected in Commitment 6 [2] and measured by this study. Thus, using the wording of the Political Declaration that includes those in need of social protection benefits, the social protection target might have been achievable. Social protection coverage would have been assessed only among a smaller group of people living with at risk of or affected by HIV in need of social protection benefits; not all of them. Such sub-analysis may still have to be conducted by governments to situate the results in their contexts and identify areas for policy actions. In 2021, UNAIDS revised the social protection target to 45%. The downward revision by UNAIDS of the HIV and social protection target to 45% of people living with, at risk of and affected by HIV and AIDS, have access to one or more social protection benefits by 2026 [6] is appropriate. Social protection coverage rates were low among the countries in our study, which are also among the most HIV affected countries of the world.

We found that the proportions of AGYW, SW, MSM and PLHIV who reported receiving social protection benefits in Malawi and Zambia did not differ from those not in these groups. One possible explanation is that community-based organisations and governments in sub-Saharan Africa have developed social protection programmes to mitigate the impact of the HIV epidemic in the general population. Households with children, girls and women have been disproportionately impacted by HIV. They are also prioritised for many social protection programmes, often with bilateral and multilateral donor support [45]. Zambia’s social protection programmes have historically focused on households with OVC expanding the eligibility criteria to include other vulnerable households such as women-headed households and those with members unable to work. Malawi's social protection programme has focused on the poorest households motivated by the need to reduce poverty and vulnerability [45]. In this study, AGYW, SW, MSM, and PLHIV may be living in households receiving social protection benefits and may report receiving the benefits.

However, in Eswatini and Tanzania, the proportions of AGYW, SW, MSM and PLHIV reported receiving social protection benefits differed between people not in these groups. In Eswatini, more AGYW reported receiving social protection benefits than older women aged 25–59. One explanation is that Eswatini’s social safety nets have included school-going children and adolescents [46]. Fewer MLHIV than men not living with HIV in Eswatini reported receiving social protection benefits. Several factors play a role in this disparity. One reason is that more men living with HIV are mobile populations (i.e., seasonal workers, transport operators, construction workers, long-distance truck drivers and uniformed forces). These mobile populations have been identified by the government of Eswatini as crucial drivers of the HIV epidemic [47]. They may not be at home often to receive social protection benefits or considered in need of social protection benefits. In Tanzania, a higher proportion of PLHIV (male and female) reported receiving social protection benefits than people not living with HIV, reflecting the inclusion of PLHIV in the Productive Social Safety Net (PSSN), the country's flag social protection programme. The PSSN, like other social protection programmes in sub-Saharan Africa, evolved in the context of HIV to alleviate the impact of HIV on orphan and vulnerable children and their caregivers [45]. However, more FSW reported receiving social protection benefits than females who were not sex workers. FSW in Tanzania might have successfully organized themselves to access and provide social protection benefits to each other [48]. At the same time, SW and MSM may face stigma and discrimination related to their social identity. They are also criminalised in many countries, which creates barriers to accessing services that could lead to disclosing their social identities [47, 48]. SW may be poor and yet not eligible for government-provided social protection or economic support to small businesses [47, 49].

The third result from our study was that a larger proportion of PLWHIV, AGYW and OVC groups in Eswatini reported receiving social protection benefits than in Malawi, Tanzania and Zambia. This result is backed by evidence and suggests that a country's income level plays an essential role in more people receiving social protection benefits [8]. A prosperous country is more likely to provide social protection benefits, including to PLHIV. Eswatini's per capita GDP is three times that of Tanzania and Zambia and nine times that of Malawi. Spending 1.31% of its GDP, Eswatini fully funded its social assistance programmes; Malawi did not. Malawi spent only 0.41% of its GDP on social assistance programmes [8]. More of Malawi's people may have depended on limited social assistance, typical among developing countries. Thus, a relatively higher proportion of Malawians reported receiving social protection benefits than the country's income would suggest [8].

The size of the HIV epidemic and the effectiveness of the HIV response play a role in linking people to social protection benefits. Eswatini outperforms Malawi, Tanzania and Zambia on the HIV testing and treatment cascade and has fewer estimated PLHIV. Eswatini's impressive AIDS response is credited, in part, to an effective multi-sectoral strategy coordinated from the Prime Minister's office by the National Emergency Response Council on HIV/AIDS (NERCHA). NERCHA also directly delivers social protection benefits, including school feeding, food distribution and social services. NERCHA is involved in decision-making about OVC educational grants, supplementary feeding, fee-waivers, agriculture input subsidies, and old age grants delivered by ministries of education, health, agriculture, and others. Moreover, Eswatini's social protection strategies directly include people living with, at risk of and affected by HIV as primary beneficiaries [46]. It has integrated HIV and social protection services within the government. As a result, Eswatini may have had more success linking people living with, at risk of, or affected by HIV to social protection benefits than Malawi, Tanzania and Zambia. However, MLHIV, may lose out on the benefits, even in relatively richer countries. Focused efforts may be required to enhance access to social protection benefits of all people living with, at risk of or affected by HIV.

To our knowledge, our study is the first to estimate social protection coverage among PLHIV, SW and MSM. We used nationally representative data sets from four countries, enabling us to compare the estimates of social protection coverage among seven sub-populations and the general population in four high HIV prevalence countries. United Nations Children's Fund (UNICEF) developed and piloted social protection questions for indicator SDG 1.3.1 in Kenya (2014), Zimbabwe (2015), Vietnam (2015) and Belize (2015), and showed that the questions worked well. UNICEF assessed the adequacy, clarity, and relevance of the questions for various population groups and settings [50]. UNICEF did not estimate social protection coverage for PLHIV, SW and MSM. We documented a methodology in this article to measure Commitment 6 and included SAS code for easy use with PHIA data sets containing HIV-related sub-population groups and social protection variables (Appendix 2).

Other nationally representative surveys measure access to social protection benefits. However, few also include HIV testing or questions relevant to identifying belonging to relevant sub-populations groups. The Multiple Indicator Cluster Survey (MICS) is one such survey. It has been periodically conducted in more than 100 low- and middle-income countries by UNICEF to assess children and women's well-being. Like the PHIA, MICS are nationally representative surveys administered to individuals in households. The MICS 6 survey asks several questions about social protection, PLHIV, AGYW and OVC. The MICS 6 survey data sets have been released for Zimbabwe, Lesotho, the Democratic Republic of the Congo and Punjab province in Pakistan. The MICS survey does not ask questions that allow respondents to identify as MSM or SW [51]. Demographic and Health Surveys (DHS) are also nationally representative cross-sectional surveys that include HIV testing and identify the various population groups of interest. Although DHS surveys have been conducted in 90 countries, allowing for significant cross-country comparisons, they unfortunately do not capture information on social protection. Neither do they capture information on MSM [52].

Other sources explored that capture social protection coverage estimates in countries included the World Social Protection Database, hosted by the ILO. The database compiles and disseminates social security data by country and population group. It presents the proportion of the population “receiving at least one contributory or non-contributory cash benefit, or actively contributing to at least one social security scheme” among children, mothers with newborns, persons with severe disabilities, unemployed, older persons, vulnerable persons and the poor [53]. Another is the World Bank’s Atlas of Social Protection Indicators of Resilience and Equity, which compiles global social protection and labour indicators. None of the two capture HIV-related information [34].

There are several limitations to our study. First, receipt of social protection benefits is self-reported, linked to a household and could not be verified independently. Respondents reporting that they or their households received benefits does not confirm that the respondent specifically received the benefit. However, it is assumed that household members shared the benefits a household received. Second, the sample sizes available to estimate the proportion of SW and MSM who reported receiving social protection benefits is small, limiting the precision of our analyses. Third, the PHIA data sets that included HIV status and social protection information were only publicly available for Eswatini, Malawi, Tanzania, and Zambia at the time of this analysis, limiting estimates outside these countries. Fourth, the social protection questions asked in our study does not distinguish between formal and informal support. Among OVC, the social protection benefits received included medical, emotional, material, social and school support. Although emotional support falls under social services and may be offered to social protection beneficiaries, it may not strictly fit in the ILO and World Bank definitions of social protection. Thus, our social protection coverage estimates may not be directly comparable to those of the World Bank and the ILO. Last, the PHIA data sets may not effectively capture receipt of social protection benefits for SW and MSM who have no fixed residence or did not identify as such or feel comfortable disclosing their social identity. People in prison, in the military, hospital, boarding schools and other institutions are not included in household-based surveys. We recommend that surveys being conducted among key populations include questions to capture social protection coverage.

## Conclusions

This study measured UNAIDS Fast-Track Commitment 6, “Ensuring that by 2020, 75% of people living with, at risk of or affected by HIV benefit from HIV-sensitive social protection.” In some of the countries with the highest HIV prevalence countries in the world (Eswatini, Malawi, Tanzania, and Zambia), access to social protection benefits among the general population, PLHIV, AGYW, OVC, SW and MSM was lower than the global average of 45% and far short of 75% indicated in Commitment 6. In Malawi and Zambia, social protection coverage among OVC, AGYW, SW, MSM, and people living with HIV (PLHIV) was similar to the general population. In Eswatini, more AGWY reported receiving social projection benefits than older women but more men not living with HIV reported receiving social protection benefits than MLHIV. In Tanzania, more PLHIV than people not living with HIV, and FSW than women who were not sex workers reported receiving social protection benefits. Including SW, MSM and other key populations in population-based surveys that measure HIV and social protection is required to better estimate the prevalence of social protection benefits for these population groups. Data on access to social protection benefits by people living with, at risk of or affected by HIV are needed to better estimate their social protection coverage.

## Supplementary Information

Below is the link to the electronic supplementary material.

Supplementary file1 (DOCX 15 kb)

Supplementary file2 (DOCX 19 kb)

## Data Availability

PHIA Project website at https://phia-data.icap.columbia.edu/datasets.

## Code Availability

Appendix 2. SAS Code.
