# Supplementary material for: Access to Social Protection by People Living with, at Risk of, or Affected by HIV in Eswatini, Malawi, Tanzania, and Zambia: Results from Population-Based HIV Impact Assessments
Source: AIDS Behav. 2022 Mar 22;26(9):3068–78. doi: 10.1007/s10461-022-03645-1 (PMC8938650; doi:10.1007/s10461-022-03645-1)
Supplement: Supplementary file 1 — Supplementary file1 (DOCX 15 kb) [file 10461_2022_3645_MOESM1_ESM.docx]

**Appendices**

**Appendix 1. Key variable names and questions used (PHIA survey instruments)**

| **Key variable names and questions used (PHIA survey instruments)** | |
| --- | --- |
| **Variable** | **Questions used** |
| **Parent sick** |  |
| momsick | Has [HHRNAME*]'s natural mother been very sick for at least 3 months during the past 12 months, that is she was too sick to work or do normal activities? YES 1 NO 2 DON'T KNOW |
| dadsick | Has [HHRNAME*]'s natural father been very sick for at least 3 months during the past 12 months, that is he was too sick to work or do normal activities? YES 1 NO 2 |
|  |  |
| **Sex work** |  |
| sellsx12mo | In the last 12 months, have you sold sex for money? ( YES 1 NO 2 DON’T KNOW ‐8 REFUSED) |
|  |  |
| **Gender of the last three sexual partners** |  |
| partgender1 | Is [INITIALS Partner 1 ] male or female? (MALE 1, FEMALE 2, DON’T KNOW ‐8 REFUSED) |
| partgender2 | Is [INITIALS Partner 2 ] male or female? (MALE 1, FEMALE 2, DON’T KNOW ‐8 REFUSED) |
| partgender3 | Is [INITIALS Partner 3 ] male or female? (MALE 1, FEMALE 2, DON’T KNOW ‐8 REFUSED) |
|  |  |
| **Economic Support** econsup03 | Has your household received any of the following forms of external  economic support in the last 12 months? Read the responses aloud, select all that apply. |
|  | Nothing |
|  | Cash Transfers (e.g. Pension, Disability Grants, Child Grant) |
|  | Assistance for School Fees, |
|  | Material Support for Support for Education (e.g. Uniforms, School Books, Education, Tuition Support, Bursaries), |
|  | Income Generations Support in Cash or Kind (e.g. Agricultural Inputs), |
|  | Food Assistance provided at the Household or external support, |
|  | Material or financial support for shelter, |
|  | social pension. |
|  |  |
| **Child support** | In the last 12 months, has your household received any… |
| supportschol12 | Support for [HHRNAME*]'s schooling, such as allowance, free admission, books, or supplies, for which you did not have to pay? (YES 1 NO 2 DON'T KNOW) |
| supportsocial12 | Social support for [HHRNAME*] such as help in household work,  training for a caregiver, or legal services, for which you did not have to  pay? (YES 1 NO 2 DON'T KNOW) |
| supportmater12 | material support for [HHRNAME*], such as clothing, food, or financial  support, for which you did not have to pay? (YES 1 NO 2 DON'T KNOW) |
| supportemot12 | emotional or psychological support for [HHRNAME*], such as  companionship, counseling from a trained counselor, or spiritual support, which you received at home and for which you did not have to pay? (YES 1 NO 2 DON'T KNOW) |
| supportmed12 | medical support for [HHRNAME*], such as medical care, supplies or  medicine, for which you did not have to pay? (YES 1 NO 2 DON'T KNOW) |
