# Supplementary material for: Access to Social Protection by People Living with, at Risk of, or Affected by HIV in Eswatini, Malawi, Tanzania, and Zambia: Results from Population-Based HIV Impact Assessments
Source: AIDS Behav. 2022 Mar 22;26(9):3068–78. doi: 10.1007/s10461-022-03645-1 (PMC8938650; doi:10.1007/s10461-022-03645-1)
Supplement: Supplementary file 2 — Supplementary file2 (DOCX 19 kb) [file 10461_2022_3645_MOESM2_ESM.docx]

**Appendices**

**Appendix 2. SAS Code**

*****************************************************************************

Program assumes that the data for each country lives in a separate library named <abbr>pubs, and that an output library named out has also been assigned

*****************************************************************************

*********************************

Create adult data set

*********************************;

**%macro** adults(abbr = , survey = );

%if &abbr = cam or &abbr = civ %then %let econvar = econsup12;

%else %if &abbr = swa %then %let econvar = econsup3;

%else %let econvar = econsup3 econsup12;

data indhh&abbr.;

merge &abbr**.p**ubs.&survey.hh (in = a keep = householdid &econvar.)

&abbr**.p**ubs.&survey.adultind (in = b where = (indstatus = **1**));

by householdid; if a and b;

if gender = **2** and sellsx12mo = **1** then fsw = **1**; else fsw = **0**;

if gender = **1** and sellsx12mo = **1** then msw = **1**; else msw = **0**;

%if &abbr. = swa %then %do; if econsup3 NOT in("A", "Z", "")then socpro = **1**; else if econsup3 = "" then socpro = **.**; else socpro = **0**;

%end; %else %do;

if econsup12 NOT in("A", "Z", "") then socpro = **1**; else if econsup12 = "" then socpro = **.**; else socpro = **0**;

%end;

if gender = **1** and partgend1 = **1** then msm = **1**;

else if gender = **1** and partgend2 = **1** then msm = **1**;

else if gender = **1** and partgend3 = **1** then msm = **1**; else msm = **0**;

if gender = **.** then msm = **.**; if **15** <= age <= **24** and gender = **2** then agyw = **1**;

else agyw = **0**; run;

data out.&survey.adultsocpro;

set indhh&abbr.;

where socpro ne **.** and age le **59**;run;

**%mend**;

%***adults***(abbr=zam, survey = zamphia2016);

%***adults***(abbr=mal, survey = mphia2015);

%***adults***(abbr=swa, survey = shims22016);

%***adults***(abbr=tan, survey = this2016);

*********************************

Create child data set

*********************************;

**%macro** kids(abbr = , survey = );

data indkids&abbr.0;

set &abbr**.p**ubs.&survey.adultind

keep = country age gender householdid personid indstatus intwt:

supportschol12 supportsocial12 supportmater12

supportemot12 supportmed12)

&abbr**.p**ubs.&survey.childind

(keep = country age gender householdid personid indstatus intwt:

supportschol12 supportsocial12 supportmater12 supportemot12 supportmed12);

where age ge **0** and age le **17**; run;

proc sort data = indkids&abbr.0 out = indkids&abbr.; by householdid personid;

run;

data indkidshh&abbr.;

merge indkids&abbr. (in = a)

abbr**.p**ubs.&survey.hh (where = (hhstatus = **1**));

by householdid;

if supportschol12 = **1** then socpro = **1**; else if supportsocial12 = **1** then socpro = **1**; else if supportmater12 = **1** then socpro = **1**;

else if supportemot12 = **1** then socpro = **1**;

else if supportmed12 = **1** then socpro = **1**; else if supportschol12 = "" and

supportsocial12 = "" and supportmater12 = "" and supportemot12 = "" and

supportmed12 = "" then socpro = **.**; else socpro = **0**;

if a; run;

data out.&survey.ovc;

set indkidshh&abbr.;

where socpro ne **.**;

run;

**%mend**;

%***kids***(abbr=zam, survey = zamphia2016);

%***kids***(abbr=mal, survey = mphia2015);

%***kids***(abbr=swa, survey = shims22016);

%***kids***(abbr=tan, survey = this2016);

*************************************

Generate estimates for each country

*************************************;

**%macro** estimates(abbr = , survey = );

%if &survey = mphia2015 %then %let maxrepw = 250;

%if &survey = zamphia2016 %then %let maxrepw = 253;

%if &survey = shims22016 %then %let maxrepw = 141;

%if &survey = this2016 %then %let maxrepw = 257;

proc surveymeans data = out.&survey.adultsocpro varmethod = jackknife;

class socpro;

var socpro;

domain gender*hivstatusfinal msm fsw msw agyw;

repweight intwt001-intwt&maxrepw. / jkcoef = **1** df = **25**;

weight intwt0;

ods output statistics = &abbr.popadult0 domain = &abbr.domadult0;

run;

%if &abbr. ne les %then %do;

proc surveymeans data = out.&survey.ovc varmethod = jackknife;

class socpro;

var socpro;

repweight hhwt001-hhwt&maxrepw. / jkcoef = **1** df = **25**;

weight hhwt0;

ods output statistics = &abbr.ovc ;

run;

%end;

**%mend**;

%***estimates***(abbr=zam, survey = zamphia2016);

%***estimates***(abbr=mal, survey = mphia2015);

%***estimates***(abbr=swa, survey = shims22016);

%***estimates***(abbr=tan, survey = this2016);

***************************************

Survey adjusted chi-square for socpro

and inclusion in each subpop by gender

***************************************;

**%macro** chisq(survey = );

%if &survey = mphia2015 %then %let maxrepw = 250;

%if &survey = zamphia2016 %then %let maxrepw = 253;

%if &survey = shims22016 %then %let maxrepw = 141;

%if &survey = this2016 %then %let maxrepw = 257;

data work.&survey.adultsocpro;

set out.&survey.adultsocpro;

if hivstatusfinal = **99** then hivstatusfinal = **.**;

run;

proc surveyfreq data = &survey.adultsocpro varmethod = jackknife;

where gender = **1**;

tables gender*hivstatusfinal*socpro gender*msm*socpro gender*msw*socpro

/ row chisq ;

repweight intwt001-intwt&maxrepw. / jkcoef = **1** df = **25**;

weight intwt0;

ods output crosstabs = &survey.maletabs0 chisq = &survey.malechisq0;

run;

proc surveyfreq data = &survey.adultsocpro varmethod = jackknife;

where gender = **2**;

tables gender*hivstatusfinal*socpro gender*agyw*socpro gender*fsw*socpro

/ row chisq ;

repweight intwt001-intwt&maxrepw. / jkcoef = **1** df = **25**;

weight intwt0;

ods output crosstabs = &survey.femaletabs0 chisq = &survey.femalechisq0;

run;

**%mend**;

%***chisq***(survey = mphia2015);

%***chisq***(survey = zamphia2016);

%***chisq***(survey = shims22016);

%***chisq***(survey = this2016);
